# Impact of Virtual Reality–Based Group Activities on Activity Level and Well-Being Among Older Adults in Nursing Homes: Longitudinal Exploratory Study

**DOI:** 10.2196/50796

**Published:** 2024-03-29

**Authors:** Yijun Li, Carlotta Wilke, Irina Shiyanov, Beate Muschalla

**Affiliations:** 1 Department of Psychotherapy and Diagnostics Technische Universität Braunschweig Institute of Psychology Braunschweig Germany; 2 VirtuaLounge GmbH Braunschweig Germany

**Keywords:** virtual reality, group activity, aging care, older adults, meaningful activity, mental health, well-being, social interaction, psychosocial capacities, activity of daily living

## Abstract

**Background:**

In addition to illness, inactivity is a risk factor for high mortality in nursing homes. Using innovative technology, such as virtual reality (VR), for meaningful group activities could provide new opportunities for solving this problem. VR interventions have already been approved as a promising method for enhancing the health of older adults.

**Objective:**

In this study, we examined whether VR-based group activities can have a positive impact on activity level and group interaction among older adults living in nursing homes.

**Methods:**

We conducted a longitudinal study and provided VR interventions as a group activity once a week for 4 consecutive weeks in nursing homes. Participants were recruited based on the experience of the nursing staff members and the natural decisions of the older adults. Within a virtual cottage, designed according to the needs of the target group, older adults were able to perform daily tasks that they were no longer able to do in real life, such as gardening and making pizza. Overall, 2 psychologists measured the psychosocial capacities, activities of daily life, and well-being before and after the interventions using standardized instruments.

**Results:**

The results focus on a total of 84 older adults from 14 nursing homes who completed at least 3 VR interventions. The results indicate that several psychosocial capacities among the older adults improved, including adherence to regulations (*P*<.001; η*²*=0.122), flexibility (*P*<.001; η*²*=0.109), and group integration (*P*<.001; η*²*=0.141). Problems related to competence also showed a slight decrease (*P*=.04; η*²*=0.039). In addition, the VR intervention promoted their proactivity (*P*<.001; η*²*=0.104) and mobility (*P*=.04; η*²*=0.039). During the VR group intervention, older adults’ well-being could be maintained at a high level. The results highlight the beneficial effects of VR intervention as a meaningful activity in nursing homes, showcasing the potential of VR applications in this setting.

**Conclusions:**

This study provides a novel and naturalistic perspective, offering new insights into the use of VR in nursing homes. The VR intervention was well accepted and fulfilled the aim of enhancing capacity and well-being. It could be a meaningful group activity in nursing homes to improve social group interaction. To provide stronger evidence, randomized controlled trials are necessary.

## Introduction

### Background

As a result of demographic changes and the development of physical and mental illnesses in the older adult population, residing in a nursing home for assisted living is a commonly chosen solution in later life. The perspectives of older adults residing in nursing homes are characterized by a prevailing sense of awaiting death and a dearth of activities that foster a sense of purpose and fulfillment [1]. Vossius et al [2] conducted a longitudinal study involving 690 older adults living in nursing homes over a span of 3 years. The median survival time in nursing homes was approximately 2.2 (95% CI 1.9-2.4) years. The annual mortality rate was approximately 30% [2,3].

On the one hand, the high mortality rate can be explained by the baseline health situation and comorbidity of the residents [3,4]. On the other hand, there is a loss of activities of daily living (ADLs). It is possible to reduce the risk of mortality by improving ADLs to maintain physical functioning [2]. ADLs encompass essential daily activities and mobility, such as eating and using the toilet. Ouden et al [5] observed a significant number of inactive older adults in nursing homes. Most of them were observed to be in a lying or sitting position [5]. A considerable proportion (67%) of the older population engaged in sedentary behavior for >8.5 hours per day [6]. This sedentary behavior and lack of communicative activity have critical implications for the prevention of physical, psychological, and social health problems [7]. This phenomenon also indicates social isolation and loneliness among older adults in nursing homes [8,9]. Older adults in nursing homes tend to be lonelier than community-dwelling older adults, even though they are often surrounded by other residents and caregivers [9-11]. Connecting with individuals with varying cognitive fitness levels is challenging in nursing home settings [10].

Several studies have been conducted regarding how loneliness and social isolation in nursing homes negatively affect mental and physical health, well-being, and mortality [12-15]. Studies have consistently shown that both loneliness and social isolation are associated with various mental health issues, including depression, feelings of hopelessness, and cognitive impairment [16,17]. Older adults residing in nursing homes experienced elevated levels of loneliness and anxiety during the COVID-19 pandemic compared to those receiving home care [18]. Overall, 69% of older individuals in nursing homes reported feelings of loneliness and 63% reported anxiety. On the other hand, among those receiving home care, 53% reported loneliness and 47% reported anxiety [18]. In addition, these conditions have been linked to impaired motor function, cardiovascular health problems, disrupted sleep, and increased frailty [13,19]. Zhao et al [20] found that higher engagement in activities was associated with lower levels of loneliness and frailty among older adults in nursing homes. The authors emphasized the importance of developing strategies to increase social and activity engagement in this population. Higher levels of activity engagement and meaningful relationships have been linked to greater satisfaction, well-being, and quality of life [15,21,22].

Therefore, there is a need to develop strategies that focus on improving ADLs, promoting engagement in activities, and enhancing social interactions among nursing home residents. These strategies have the potential to enhance overall well-being and quality of life and potentially reduce loneliness, social isolation, and mortality among older individuals residing in nursing homes [1,20,23].

Enhancing activity and social interaction among older adults can be effectively supported through meaningful daily group activities [24]. Participating in group activities fosters a sense of belonging, promotes social engagement, and contributes to overall well-being [24]. It provides opportunities for increased social interaction with fellow residents and emotional support through participating in games and identifying with teams [24]. It is also important that these activities are “meaningful” to the residents. Research by Tak et al [25] demonstrated that if activities are not relevant or meaningful to the residents, they may prefer to do nothing or passively watch television. Meaningful group activities are described as those that hold significance or provide enjoyment for individuals, aligning with their current and past interests, routines, habits, and roles and improving their mental or physical function [26-29]. It has been shown that meaningful activities enhance social engagement and well-being and reduce loneliness among older adults living in nursing homes [30]. Nevertheless, there are several barriers to providing meaningful group activities in nursing homes. One major challenge is the shortage of personnel. Nursing homes are already facing difficulties in filling nursing home positions due to a shortage of skilled workers [31], and this is expected to persist and worsen in the coming years. Insufficient staffing limits the capacity to organize several meaningful activities [32,33]. In addition, there may be constraints related to limited space and equipment within nursing homes [33]. Therefore, there is a need to develop new, low–resource dependent, easily applicable, meaningful group activities [34,35]. Moreover, the demands of older adults in need of care are evolving, including their expectations regarding the technical equipment in nursing homes. On the basis of a population survey conducted in Germany between 2009 and 2014, only a small percentage of older adults aged >65 years used smartphones, but by 2019, more than half of them were already using these devices. In addition, internet use has also experienced significant growth since 2009, with 74% of the older adult population using the internet in 2019 [35]. Therefore, introducing innovative, technology-based interventions such as virtual reality (VR) [36] for group activities could be a promising solution to improve social connections and activities for older adults in nursing homes [12,37,38].

Fully immersive VR has emerged as a feasible method of intervention in older adults’ rehabilitation and aging care [36,39]. By using head-mounted devices (HMDs) and controllers, VR technology provides users with a fully immersive experience and a sense of presence. The unique characteristics of VR offer a viable solution to the barriers faced in nursing homes [33]. These barriers include the health status of older adults, limited physical space in the environment, and organizational challenges such as staffing shortages and funding constraints. First, VR proves to be accessible and accommodating for older adults with limited mobility. For example, individuals can remain seated in a wheelchair while experiencing the sensation of being on a mountain surrounded by stars. This enables older adults to engage in activities that would otherwise be physically challenging or impossible. Moreover, VR interventions ensure safety as they eliminate environmental risks and can be paused at any moment, which is particularly important for populations considered vulnerable such as older adults [40]. Second, VR interventions are flexible and require minimal physical space, similar to the space requirements for small-group activities [41]. This flexibility empowers the staff members to efficiently organize activities, while also reducing the costs associated with transportation. Moreover, the effectiveness of VR interventions for older adults has been demonstrated. The immersive and presence-inducing nature of VR has shown evidence comparable to that of traditional interventions in mental health [42]. Using VR as a medium to improve ADLs for older adults has already shown promising effects [43], and it was found to be effective in reducing loneliness and social isolation [44-47]. In the field of mental health, VR is considered an effective method for training and therapy for cognitive functions and for enhancing the well-being of older adults [48-50]. In addition, several studies have reported high acceptance of VR experiences among older adults [39,51,52].

However, recent interventions have primarily focused on singular concepts such as training or entertainment, and there is a lack of studies exploring VR interventions as daily group events in nursing homes that provide opportunities for older adults to connect and interact socially. In this study, we developed and evaluated a series of VR interventions, aiming to enhance the overall health and well-being of older adults in nursing homes. The VR interventions combine training activities and entertainment to create meaningful group ADLs in nursing homes. As discussed previously, meaningful activities should align with older adults’ current and past interests, routines, habits, and roles [28,29]. Studies have shown that older adults who engage in daily and household activities experience less decline in mobility [53]. For example, gardening has been recognized as a promising activity for reducing loneliness and improving socialization [54,55]. Building upon these findings, our VR group intervention focuses on providing older adults in nursing homes with virtual environments that allow them to engage in daily tasks they may no longer be able to perform, such as baking a pizza, handcrafting, and gardening. Through these simulated activities, older adults have the opportunity to experience the fulfillment of completing familiar tasks, while preserving and enhancing their physical and mental functioning. By using tasks that are familiar to them, we aimed to reduce fear or demotivation, which might occur when being confronted with new technology. The meaningful activities chosen for the VR game offer older adults an enjoyable experience and contribute to their overall well-being. Organizing these daily activities in a virtual environment incurs lower costs in terms of time and equipment compared to real-life implementation. In addition to the VR scenario, we developed an aid system and an automated program that enable staff members to easily facilitate VR group sessions with older adults.

In summary, this exploratory study investigated the effectiveness of implementing VR interventions as meaningful group activities for older adults in nursing homes. The primary focus was on evaluating the older adults’ activity and mobility levels, well-being, social interaction, and mental capacities over the course of a 4-week VR group intervention. By supporting well-being and psychosocial capacities, these interventions have the potential to address key challenges faced by older adults in nursing homes.

### Research Question and Hypotheses

This observational intervention study examines the following question: Does VR-based group activity have any positive impact on the daily lives of older adults living in nursing homes? The following hypotheses were tested:

- Hypothesis 1: Over the course of a 4-week VR intervention, psychosocial capacities and ADLs of older adults in nursing homes will remain stable or even improve.
- Hypothesis 2: Over the course of a 4-week VR intervention, older adults’ well-being will remain stable or even improve.

## Methods

### Study Design

This longitudinal study using pre-post measures was conducted in naturalistic settings in nursing homes in a city with 250,000 inhabitants in Germany. After contacting all 31 local nursing homes, a total of 15 (48%) nursing homes chose to participate in the project.

### Selection of Participants

We contacted all nursing homes by telephone. Subsequently, those nursing homes expressing interest were provided with a comprehensive briefing via email outlining the selection criteria. Participants were selected from the nurses in the nursing homes, who considered both the basic data (eg, medical history) about the older adults from the nursing home information system and their extensive experience in assisting older adults with their daily living, while also assessing the older adults’ willingness to participate. The selection criteria were as follows: (1) older adults were aged >60 years; (2) they had at least 1 arm and 1 hand that they were able to use (this ensures their interaction with the virtual environment); (3) they were still able to see and hear, and the use of audio aids or glasses was permitted; (4) they were able to participate in an oral interview with the researchers and did not have severe dementia; and (5) they did not have diseases, such as epilepsy, that are contraindications for VR activities.

We set a control group that underwent the same measurement procedure as the intervention group, except that they did not undergo the VR intervention phase. The control group participants were chosen based on the advice from the nursing staff members. The older adults in the control group had a low willingness to participate in the VR intervention and expressed a preference for interviews. Of the 15 nursing homes, 1 (7%) chose to solely participate in the control group due to low willingness to organize new group events and expressed a preference for participating in the project only through interviews with older adults.

### Procedure and Data Collection

The interventions were conducted for approximately 3 months in each participating nursing home. During this period, the intervention group went through 3 phases: premeasurement phase, 4 weeks of VR group intervention, and postmeasurement phase (Table 1). During the premeasurement phase, each older adult was offered an individual initial appointment. During this appointment, a psychologist explained the schedule, privacy agreement, and consent form to the older adult. One week after the initial appointment, the older adult underwent the first pretest interview (T0; baseline) with a psychologist. This baseline assessment covered mental capacity, ADLs, and well-being. In the following week, a brief, second pretest (T1) was conducted to familiarize the older adults with the well-being assessment. This served as a warm-up assessment and was repeated after each subsequent intervention session. The intervention phase began after the premeasurement phase. Group interventions were conducted every week for 4 weeks (T2-T5). These sessions were facilitated by a project psychologist and a technical assistant. Their role was to introduce the older adults to the VR program and provide support throughout the VR sessions. There were always 3 to 5 older adults in a group for an intervention. During each VR intervention session, the older adults were presented with tasks to solve in a virtual environment. After completing the tasks, the older adults participated in a 3-minute virtual tour of a landscape to relax. Following each VR group session, the older adults’ individual well-being was assessed. One week after completing all 4 VR group interventions, the same psychologist who conducted the pretest interviews assessed the older adults’ mental capacities, ADLs, and well-being in the posttest phase through a postintervention interview (T6). A follow-up interview (T7) with the same content was conducted 3 weeks after the posttest phase to assess the stability of the posttest results.

**Table 1:** Procedure of the virtual reality (VR) group intervention study in a nursing home (as per the focus of this paper)^a^.

	Week 1	Week 2	Week 3	Week 4	Week 5	Week 6	Week 7	Week 10
Event	Preintervention assessment 1 (T0)	Preintervention assessment 2 (T1)	VR activity 1 (T2)	VR activity 2 (T3)	VR activity 3 (T4)	VR activity 4 (T5)	Postintervention assessment 1 (T6)	Follow-up (T7)
Content	Baseline demographic information Mini-ICF-APP^b^ ADL^c^ WHO-5^d^	Warm-up WHO-5	VR group activity WHO-5	VR group activity WHO-5	VR group activity WHO-5	VR group activity WHO-5	Postintervention interview Mini-ICF-APP ADL WHO-5	Follow-up-interview Mini-ICF-APP ADL WHO-5
Implementation	Psychologist	Psychologist	Psychologist and technical assistant	Psychologist and technical assistant	Psychologist and technical assistant	Psychologist and technical assistant	Psychologist	Psychologist

^a^Some procedures such as a questionnaire for feedback and obtaining perceptions from the older adults are not presented in this table.

^b^Mini-ICF-APP: Mini-ICF-Rating for Impairment in Psychological Activities and Capacities [56].

^c^ADL: activity of daily living [57].

^d^WHO-5: World Health Organization–Five Well-Being Index [58].

The study was conducted during the COVID-19 pandemic, and the safety of our older adult participants was our top priority. From our initial contact with the nursing homes, we inquired about and strictly adhered to the COVID-19 guidelines governing group events. Our team members underwent COVID-19 testing at public testing institutes within 24 hours before each visit to a nursing home. Even if a team member exhibited symptoms similar to those of COVID-19 but obtained a negative test result, they were not allowed to enter the nursing home as an extra precaution. During individual interviews, all team members wore masks, and regular hand disinfection was practiced throughout their stay in the nursing homes. During the VR interventions, we maintained a safe distance between the older adults, and the VR equipment was thoroughly disinfected after each use to ensure the highest level of safety for all participants.

### VR Intervention and Equipment

In collaboration with the technical company, VirtuaLounge, we developed a virtual vacation home to facilitate VR interventions for the older adults. We designed the meaningful VR activities in the virtual vacation home with an older adult–centered approach, drawing upon our understanding of older adults’ daily routines and incorporating the valuable suggestions received from older adults during the pilot program testing phase while developing the VR intervention. The central aspect was to make the VR intervention as accessible as possible to the older adult population, while minimizing the barriers to use. For example, we ensured that older adults, including those using wheelchairs, could actively participate in the entire intervention by setting up the VR experience in a sitting format. We optimized the interaction with the VR environment to be easily manageable with only 1 finger, and all the tasks were designed to be completed using only 1 hand.

**Figure 1:**
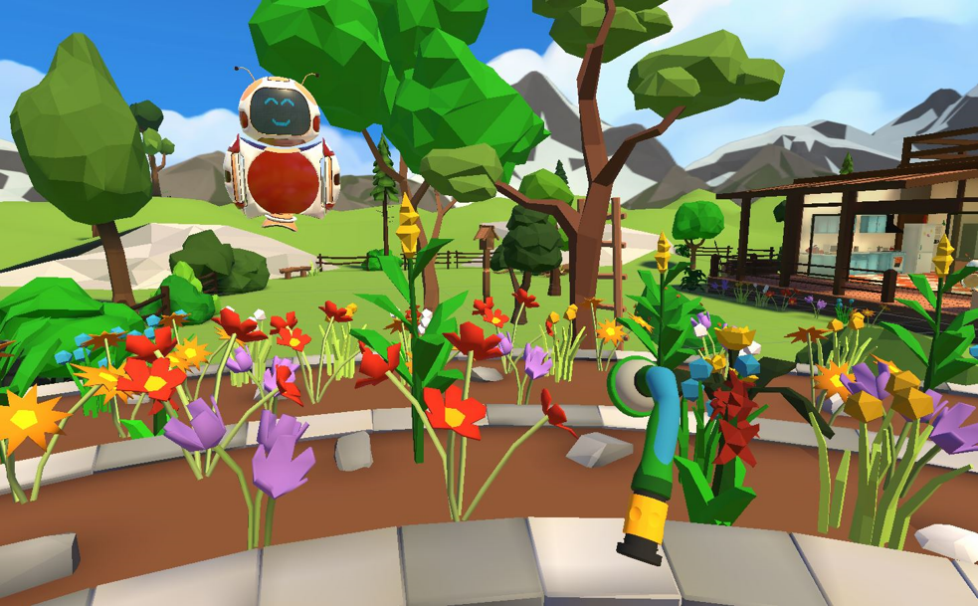
Example task—gardening.

**Figure 2:**
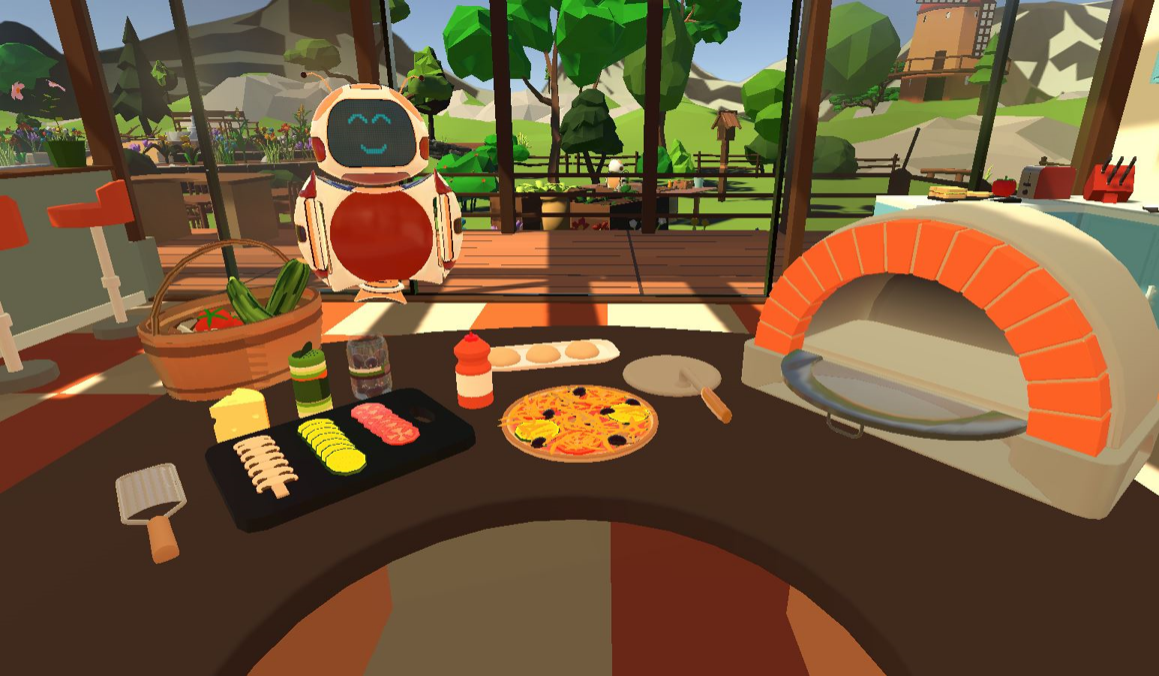
Example task—baking a pizza.

**Figure 3:**
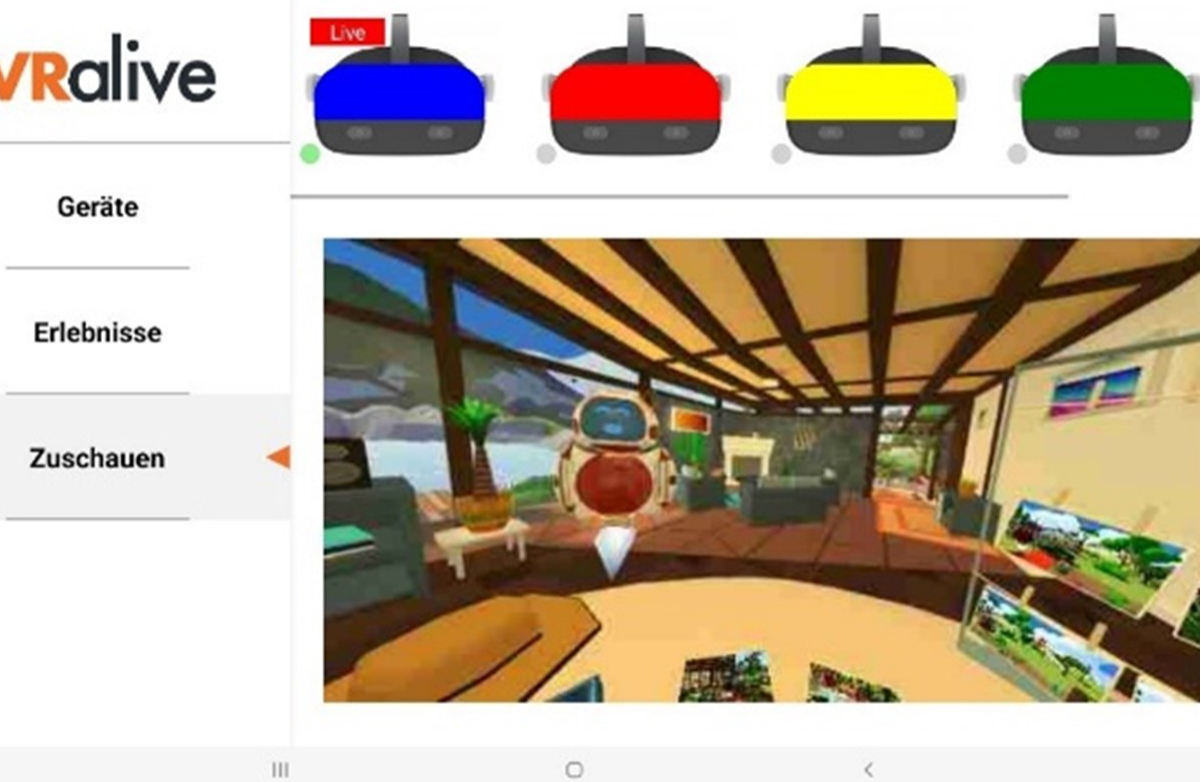
Screenshot of the tablet system.

The interventions were conducted over a period of 4 weeks in regional nursing homes, with each session lasting approximately 30 minutes. Within each VR session, participants engaged in 4 or 5 tasks that were no longer possible for them to perform in real life. These tasks were integrated into a cohesive storyline, resulting in an immersive experience for the older adults. Our storyline revolved around 4 classic settings within a vacation home: living room, crafts station, garden, and kitchen (Figures 1 and 2). The tasks encompassed various routine activities, such as building furniture, gardening, and cooking in the kitchen. We devised a tablet control system specifically for those conducting the procedures to address the challenge of assisting older individuals wearing the nontransparent HMD. This system enables remote connectivity between the tablet and HMD. Through the tablet interface, a live view of the older adults’ perspective is displayed, allowing the technical support personnel to monitor the progress of individual tasks for all participants. In addition, the tablet allows supporters to adjust the sound settings and remotely initiate or terminate the program on each HMD (Figure 3). This innovative solution enhances the ability to provide real-time assistance and control during the VR interventions.

The VR setup used in this study involved the use of the stand-alone VR Headset Pico Neo 3 Pro along with the Pico Neo 3 controller (Pico Technology Co, Ltd). The resolution of the VR headset was set at 1832 × 1920 per eye. The headset operated at a refresh rate of 72 Hz and featured 6 dfs inside-out tracking capabilities. In addition to the VR equipment, a Samsung Galaxy Tab S6 Lite (Samsung Electronics Co, Ltd) with an Android 12 operating system served as the remote tablet for the study. The system was programmed using .Net and C# programming languages.

### Instruments

Consistent with our hypotheses, *psychosocial capacities* (hypothesis 1) included the mental capacities to adapt to daily life in a nursing home, which was assessed using the Mini-ICF-Rating for Impairment in Psychological Activities and Capacities (Mini-ICF-APP) scale [56]. This scale encompasses 13 psychosocial capacities, including proactivity and mobility. *ADLs* (hypothesis 1) included physical activity and mobility of the participants, which were assessed in more depth using the ADL-Barthel Index [57] that encompasses basic ADLs. *Well-being* (hypothesis 2) was measured using the World Health Organization–Five Well-Being Index (WHO-5) [58]. These measures were administered to both the intervention group and the control group, allowing for a comprehensive evaluation of the outcomes in both groups (Table 1).

The *Mini-ICF-APP capacity rating* [56] is an established instrument for describing a person’s psychosocial capacity status. It has been translated internationally into many languages and is recommended in social medicine guidelines [59]. Among other settings, it is commonly used in settings of psychosocial rehabilitation. It is internationally recognized and has proven to be manageable, reliable, and valid in clinical practice [60-63]. In this study, the Mini-ICF-APP rating was designed to assess the psychosocial capacities for living in the nursing home, that is, performing certain basic activities on their own. The scale covers the following capacity dimensions: (1) adherence to regulations, (2) planning and structuring of tasks, (3) flexibility, (4) competence and knowledge application, (5) capacity to make decisions and judgments, (6) proactivity and spontaneous activities, (7) endurance, (8) self-assertiveness, (9) contact with others, (10) group integration, (11) intimate relationships, (12) self-care, and (13) mobility. Each dimension is rated using an 8-point rating scale (0=this is a strength of me to 7=this is impossible for me). The Mini-ICF-APP interview guide and questionnaire were adapted for the older adults by focusing on activities that individuals in need of care could still perform independently while residing in a nursing home. In a pilot study, 8 participants were interviewed by both project psychologists. Of the 2 psychologists, 1 conducted the interview, and both the interviewers completed Mini-ICF-PP rating sheet based on the responses provided by the older adults. On average, the interrater reliability over all 13 capacity dimensions was *r*=0.857. Psychosocial capacities were measured at 3 measurement time points (T0, T6, and T7).

*ADL* [57] was measured before the first VR intervention (T0), after the intervention (T6), and at a 3-week follow-up after the posttest phase (T7). It includes 10 dimensions of daily activity: (1) eating, (2) washing and showering, (3) body care, (4) dressing and undressing, (5) stool control, (6) urine control, (7) toilet use, (8) transfer from bed to chair, (9) movement and mobility, as well as (10) climbing stairs. The total score on the Activity of Daily Living-Barthel Index (ADL-BI) ranges from 0 to 100, with higher scores indicating greater independence in performing daily activities. A score of 0 indicates complete dependence on assistance for all activities, whereas a score of 100 indicates complete independence. An ADL score <80 indicates a need for care of >2 hours a day [64]. In the interview with the study participants, the psychologists asked about the activities one after the other and checked the plausibility of the answers of the older adults were. In cases of doubt, supplementary external judgments were obtained from the caregivers to ensure data validity.

The third instrument used in this study was the *WHO-5* [58], which is a concise self-report measure of current mental well-being. The assessment of well-being using the WHO-5 was conducted at baseline, after each VR intervention session, and during the postintervention and follow-up interviews. Numerous studies have demonstrated the validity of the WHO-5 as a screening tool for depressive mood and as a measure of treatment outcomes in clinical trials, and it has also shown good construct validity for assessing well-being in both younger and older populations [65,66]. The WHO-5 has been translated into >30 languages [66]. It consists of five statements that assess the individual’s (1) good mood and cheerfulness, (2) relaxation, (3) activity and energy, (4) regenerative capacity through sleep, and (5) enthusiasm. Each statement is rated on a scale ranging from 1 (“at no time”) to 5 (“all the time”). For example, 1 statement reads as follows: “Last week, I was happy and in a good mood.” These statements are straightforward and nonintrusive in nature [66]. Typically, the questionnaire covers a 14-day period; however, considering the older adult participants and the study’s weekly interventions, a 7-day period was deemed appropriate. Therefore, the assessment inquired about the individual’s well-being, relaxation, activity level, quality of sleep, and interest in life over the past 7 days. Well-being was measured at each of the 8 measurement time points (T0-T7; Table 1).

### Statistical Analyses

The data collected from the study were entered into the statistical software SPSS (IBM Corp) [67] for analysis. A repeated-measures ANOVA was conducted to analyze the data obtained from the 84 older adults in the intervention group who had participated in at least 3 interventions. The analysis focused on psychosocial capacities (Mini-ICF-APP), ADL, and well-being (WHO-5).

Owing to unequal sample sizes, a 2-factor, repeated-measures ANOVA between the intervention group and the control group could not be performed. However, the data from the control group (consisting of 11 participants) are presented descriptively, enabling a comparison with the values obtained from the intervention group.

### Ethical Considerations

This study was funded by the German Federal Ministry of Education and Research (BMBF) (project number: 16SV8561 VRalive). This study was approved by the ethics committee of Technische Universität Braunschweig (FV-2020-18). Before the study, informed consent, confidentiality, and informed data protection were obtained from the participants or their life caregivers under the supervision of nursing staff. The older adults were informed of their ability to opt out at any time. The VR activities were provided as daily activities in the nursing home, and there was no compensation provided. All activities carried out in the nursing home were in strict compliance with the current nursing home COVID-19 prevention and treatment policy. For secondary analyses using existing data, we specified that the original consent approval covers secondary analysis without additional consent. The collected data were anonymized and deidentified.

## Results

### Participants and Demographic Information

A total of 116 older adults aged ≥60 years initially participated in the VR intervention group. Of these 116 participants, 31 (26.7%) discontinued their involvement in the VR group. The primary reasons cited for dropping out were concerns related to data protection and illness or death (Table 2).

**Table 2:** Reasons for dropping out (n=31).

Reasons	Values, n (%)
No more interest, without reasons	5 (16)
Several events are occurring	4 (13)
Incapable of participating due to illness or death	12 (39)
Virtual reality–related sickness (“cybersickness”)	5 (16)
No interest in interviews	1 (3)
Alternative events are preferred	2 (6)
Leaves retirement home	1 (3)
Cancellation of the group owing to very few participants	1 (3)

Sociodemographic information was collected at baseline and included older adults’ age, sex (male, female, or intersex), and educational and working history. The intervention group (N=116) had an average age of 80.74 (SD 8.49) years. The age range at the time of the intervention varied from 60 to 97 years. There was a higher proportion of female participants (81/116, 69.8%). Most participants (107/116, 92.2%) in the intervention group had no previous experience with VR. Table 3 presents more detailed demographic information.

We decided to consider only the data from older adults who participated in a minimum of 3 VR interventions to ensure that the analysis focused on the impact of the VR intervention. This resulted in a final sample size of 84 older adults to be analyzed statistically. Hence, for the purpose of this paper, statistical evaluation will be conducted on the data obtained from 84 older adults from the VR intervention group.

Furthermore, 12 older adults participated in the control group. One of the participants discontinued due to death (this has already been accounted for in the dropout statistics, as indicated in Table 2). The age range of the control group participants spanned from 61 to 94 years, with an average age of 83.75 (SD 8.97) years, and 10 (83%) of the 12 participants were women. It is important to note that the selection of participants for the control group was based on the perceptions of the nursing staff members and the natural decisions made by the older adults themselves. Consequently, the sample size of the control group in this study is notably small, rendering it insufficient for a robust comparison with the intervention group. Therefore, detailed information about the control group, which is presented alongside the intervention group data, is provided in Multimedia Appendices 1-3. The limitations associated with the small control group sample size are discussed further in the Strengths and Limitations section.

**Table 3:** Sociodemographic data about the older adults participating in the virtual reality (VR) intervention (N=116).

Features		Values
Age (y), mean (SD; range)		80.74 (8.49; 60-97)
**Sex, n (%)**		
	Female	81 (69.8)
	Male	35 (30.2)
**Education, n (%)**		
	None	7 (6)
	Special school	2 (1.7)
	Primary school	1 (0.9)
	Secondary school	17 (14.7)
	Primary school or grade 9 or 10	79 (68.1)
	Abitur	10 (8.6)
**Professional qualification, n (%)**		
	None	37 (31.9)
	Craft profession or skilled work	67 (57.8)
	Master	7 (6)
	University studies	5 (4.3)
**Longest professional activity in working life, n (%)**		
	Craft, industry, or production	37 (31.9)
	Research and development	3 (2.6)
	Agriculture	3 (2.6)
	Office or management activities	24 (20.7)
	Service, gastronomy, or customer service	26 (22.4)
	Practical health care (nurse, physician, therapist, or similar)	10 (8.6)
	Housewife	12 (10.3)
	Missing indication	1 (0.9)
**Frequency of visits from trusted people, n (%)**		
	Several times a week	63 (54.3)
	Weekly	27 (23.3)
	Every 2-3 weeks	8 (6.9)
	Monthly	2 (1.7)
	Less frequently than monthly	2 (1.7)
	No regular contacts	14 (12.1)
**Previous experience with** **VR,** **n (%)**		
	No	107 (92.2)
	Yes	9 (7.8)

### Outcomes

Tables 4 and 5 present a summary of the results for the Mini-ICF-APP, ADL, and WHO-5 measures at specific measurement time points for both the intervention group and the control group. A macrolevel analysis indicates significant differences in the mean scores of Mini-ICF-APP (*P*<.001; η*²*=0.150) and WHO-5 (*P*=.04; η*²*=0.032) and the sum score of ADL (*P*=.02; η*²*=0.050) within the intervention group.

**Table 4:** Comparison of the older adults’ scores at baseline, at the end of the intervention, and 3 weeks after the postintervention assessment.

		Baseline (T0), mean (SD)	Postintervention assessment (T6), mean (SD)	Follow-up (T7), mean (SD)	rANOVA^a^ (n=84)	
					*P* value	*η*²
**Capacities (Mini-ICF-APP^b^)**						
	Adjustment to rules and routines	2.57 (0.88)	2.15 (1.05)	2.04 (1.08)	<.001^c^	0.122
	Planning and structuring tasks	3.13 (1.82)	2.95 (1.92)	3.13 (2.08)	.56	0.007
	Flexibility and adaptability	2.38 (0.90)	1.86 (1.19)	1.87 (1.22)	<.001^c^	0.109
	Competence and knowledge application	2.25 (1.25)	1.99 (1.55)	1.94 (1.52)	.04^d^	0.039^e^
	Capacity to make decisions and judgments	2.57 (1.12)	2.42 (1.40)	2.48 (1.35)	.57	0.007
	Proactivity and spontaneous activities	2.39 (1.19)	2.04 (1.25)	1.90 (1.26)	<.001^c^	0.104
	Resilience and perseverance	2.54 (1)	2.25 (1.18)	2.29 (1.14)	.07	0.032
	Self-assertiveness	2.60 (1.09)	2.43 (1.15)	2.40 (0.96)	.30	0.014
	Capacity to talk with and contact third parties	2.39 (1.41)	2.14 (1.35)	2.25 (1.42)	.15	0.023^e^
	Group integration	2.71 (1.39)	2.26 (1.36)	2.04 (1.21)	<.001^c^	0.141
	Capacity to form close relationships	2.61 (1.58)	2.45 (1.66)	2.49 (1.75)	.57	0.007
	Self-care and self-sufficiency	3.29 (1.76)	3.18 (1.84)	3.11 (1.86)	.63	0.006
	Mobility and transportability	2.39 (1.46)	2.37 (1.59)	2.40 (1.54)	.95	0.001
	Average score	2.60 (0.75)	2.35 (0.85)	2.33 (0.88)	<.001^c^	0.150^e^
**ADL^f^**						
	Food	9.29 (1.76)	9.46 (1.56)	9.64 (1.30)	.11	0.027^e^
	Bath	1.85 (2.43)	1.73 (2.39)	2.02 (2.47)	.42	0.010
	Washing	4.52 (1.48)	4.58 (1.39)	4.64 (1.30)	.76	0.003
	Dressing and undressing	7.20 (3.41)	7.38 (3.68)	7.32 (3.51)	.84	0.002
	Stool control	7.92 (3.74)	8.04 (3.72)	7.80 (3.75)	.83	0.002
	Urine control	6.31 (4.40)	6.55 (4.25)	6.90 (4.31)	.27	0.016^e^
	Using the toilet	8.27 (3.76)	8.63 (3.41)	9.11 (2.71)	.03^d^	0.040
	Bed or wheelchair transfer	12.74 (4.93)	13.15 (4.58)	12.98 (4.66)	.22	0.018^e^
	Movement or mobility	9.11 (4.40)	9.46 (4.39)	9.64 (4.16)	.04^d^	0.039
	Climbing stairs	4.35 (4.40)	4.76 (4.17)	4.88 (4.46)	.28	0.015
	Total score	71.55 (23.19)	73.75 (23.32)	74.94 (22.29)	.02^d^	0.050^e^

^a^rANOVA: repeated ANOVA.

^b^Mini-ICF-APP: Mini-ICF-Rating for Impairment in Psychological Activities and Capacities; the scale ranges from 0 (“clearly a strength of mine”) to 7 (“I cannot do at all”).

^c^*P*<.001.

^d^*P*<.05.

^e^The value was corrected according to Greenhouse Geisser.

^f^ADL: activity of daily living; the scale ranges from 0 to 15.

**Table 5:** Comparison of older adults’ scores regarding their well-being (World Health Organization–Five Well-Being Index [WHO-5]^a^) before the intervention (T0 and T1), during the intervention (T2-T5), after the intervention (T6), and 3 weeks after the postintervention assessment (T7).

	Time points, mean (SD)									rANOVA^b^		
	T0	T1	T2	T3	T4	T5	T6	T7	*F* test (*df*)		*P* value	*η*²
Good mood and cheerfulness	4.08 (0.95)	3.90 (1.04)	3.78 (1.13)	3.94 (1.02)	3.90 (0.99)	3.85 (1.03)	3.83 (0.98)	3.90 (1.04)	1.893 (5.51)		.09	—^c^
Relaxation	4.02 (0.01)	3.89 (1.22)	3.98 (1.09)	3.90 (0.96)	3.94 (1.04)	3.93 (1.02)	4.06 (1.09)	3.89 (1.22)	0.554 (7.00)		.79	0.008
Activity and energy	3.54 (1.27)	3.27 (1.25)	3.32 (1.33)	3.52 (1.21)	3.57 (1.18)	3.23 (1.26)	3.60 (1.11)	3.27 (1.25)	2.050 (7.00)		.048^d^	0.029
Regenerative capacity through sleep	3.49 (1.35)	3.65 (1.36)	3.71 (1.26)	4.03 (1.05)	4.03 (1.21)	3.89 (1.36)	4.01 (1.12)	3.65 (1.36)	3.867 (5.85)		.001^e^	0.054^c^
Enthusiasm	3.64 (1.17)	3.72 (1.28)	3.60 (1.17)	3.73 (1.22)	3.85 (1.22)	3.68 (1.29)	4.12 (1.12)	3.72 (1.28)	3.541 (7.00)		.001^e^	0.049
WHO-5 (total)	3.75 (0.75)	3.69 (0.83)	3.68 (0.83)	3.82 (0.79)	3.86 (0.82)	3.72 (0.84)	3.92 (0.78)	3.91 (0.87)	2.235 (5.85)		.04^d^	0.032^c^

^a^A 5-point Likert scale ranging from 0 (“at no time”) to 5 (“all the time”).

^b^rANOVA: repeated ANOVA.

^c^The value was corrected according to Greenhouse Geisser.

^d^*P*<.05.

^e^*P*<.01.

**Figure 4:**
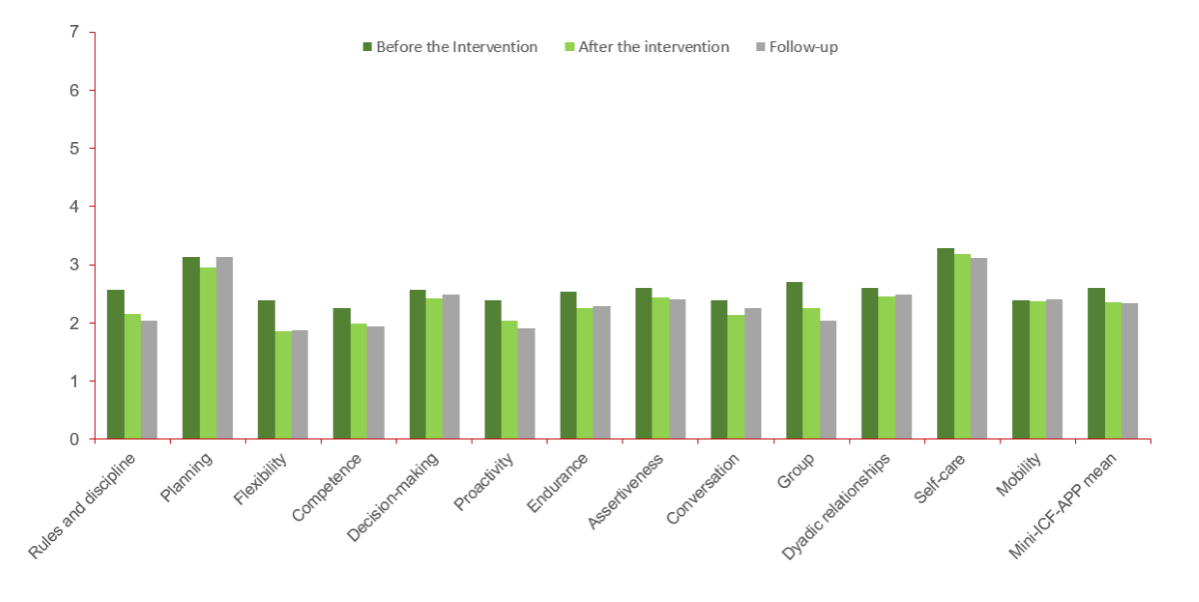
Changes in older adults’ scores for the ability dimensions (Mini-ICF-Rating for Impairment in Psychological Activities and Capacities; Mini-ICF-APP) across the measurement time points: before the intervention, after the intervention, and follow-up during the intervention.. 0=This is clearly a strength of person, 1=Person is better than many others, 2=Person can do this well, 3=Person can somehow work with this, 4=Person does not always get this to work, 5=Person has problem with this, 6=Person needs help in this regard, and 7=Person is fully unfit.

Specifically, the data from the Mini-ICF-APP indicated slight reductions in some psychosocial capacity impairments within the intervention group (Table 4 and Figure 4): adherence to regulations (*P*<.001; η*²*=0.122), flexibility (*P*<.001; η*²*=0.109), proactivity (*P*<.001; η*²*=0.104), and group integration ICF (*P*<.001; η*²*=0.141). Problems related to competence also showed a slight decrease (*P*=.04; η*²*=0.039).

Overall, 2 ADLs seemed to improve over the course of the VR intervention (Table 4): “Using the toilet” (*P*=.03; η*²*=0.040) and “mobility” (*P*=.04; η*²*=0.039).

**Figure 5:**
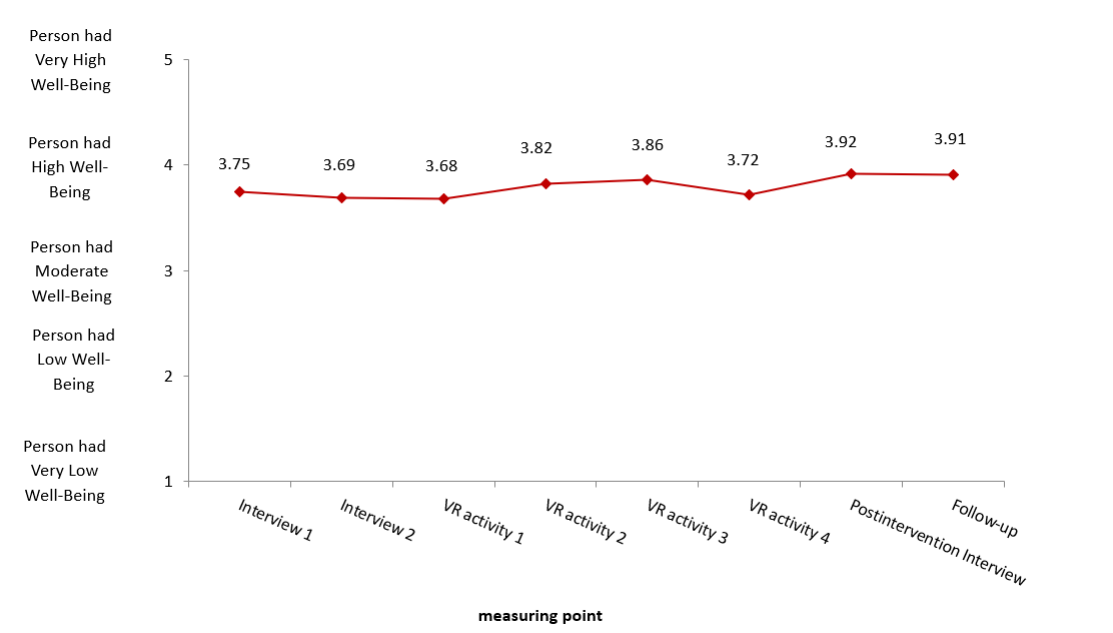
Mean of the World Health Organization–Five Well-Being Index for the intervention group at 8 weekly time points. VR: virtual reality.

In terms of well-being (WHO-5), 3 of 5 items in the WHO-5 showed significant changes (Table 5 and Figure 5): “feeling active” (*P*=.048; η*²*=0.029), “sleeping well” (*P*<.001; η*²*=0.054), and “being full of interest for life” (*P*<.001; η*²*=0.049). This indicates a slight variation in well-being over the course of the 4-week VR intervention period.

### Side Effects of VR

Throughout the VR intervention, there were no reported instances of older adults falling or accidentally colliding with nearby objects based on their sitting poses. In addition, there was no indication of potential interference with medical devices. While a few cases of cybersickness (5/31, 16%; Table 2) were reported, these were promptly addressed and resolved.

## Discussion

### Summary

The study aimed to determine whether a specifically designed VR intervention had a positive impact on nursing home inhabitants in terms of psychosocial capacities, activities, and well-being. In general, some dimensions of psychosocial capacities, activity, mobility, and well-being of older adults in the intervention group showed a slight positive improvement over the course of the intervention. Improvements were observed in adherence to regulations, flexibility, proactivity, competence, and group integration. The older adults showed improvement in their ability to use the toilet and physical mobility. The older adults reported feeling active, experiencing better sleep, and displaying greater interest in daily life.

### Principal Findings and Comparison to Previous Studies

#### Psychosocial Capacities

The results of the study demonstrate an improvement substantially in the older adults’ capacity to adhere to daily routines in the nursing home. This indicates that they have become more proficient in following schedules and maintaining self-discipline. In addition, the older adults show a greater willingness to adapt to and switch between different tasks or activities, both within the group setting and in public. Studies have shown that VR interventions can enhance the cognitive abilities of older adults, and these improvements are often correlated with their intrinsic motivation for training. This motivation can be triggered either by the engaging nature of the game itself or by the immersive experience facilitated by VR technology [68,69]. In addition, the study conducted by Fan et al [46] demonstrated that using VR as a form of entertainment for older adults can enhance their achievement motive and self-esteem, leading to improved mental health outcomes and reduced isolation, particularly among community-dwelling older adults. It is reasonable to assume that similar benefits could be experienced by older adults residing in nursing homes as well. In our case, the older adults’ intrinsic motivation may stem from their own desire to participate in meaningful VR activities and engage with others, whereas extrinsic motivation could arise from the supportive environment provided by the staff members and the positive experiences associated with the VR group activity. The combination of intrinsic and extrinsic motivation appears to have contributed to the observed improvements in discipline and flexibility among the older adults; with these improvements, the older adult could fit better with daily life in the nursing home. However, it should be acknowledged that flexibility may also have been influenced by factors such as the visits from and relationship with the VR project psychologist. Over time, as the psychologist became more familiar with the older adults, they could have corrected any initial bias in the older adults’ self-assessment, leading to more accurate evaluations based on their pragmatic experience.

The study findings indicate that the older adults’ use and retention of competence and knowledge have improved. This outcome aligns with the study’s design, which focused on implementing VR interventions with meaningful activities based on the older adults’ familiar daily tasks. The positive impact of this approach is evident in the results obtained. The older adults were able to engage with the new VR activities, thus increasing their competence. Moreover, the result of improved capability of group integration indicated that after the VR intervention, the older adults gradually developed an interest in the group activity and expressed a desire to retry the task in the following week. A previous study by Padilha et al [70] found that VR offers a learning experience from interaction with the virtual environment and enhances knowledge acquisition in nursing education. It is plausible that VR can also be a promising tool for older adults to enhance these mental capabilities. Multiple studies have demonstrated that VR interventions have a positive impact on the memory and information-processing abilities of older adults [68,71,72]. VR video games could enhance the working memory and reasoning abilities of older adults [68]. The authors also suggested that VR interventions may improve problem-solving and planning skills, which are findings that align with those of our own study.

In summary, the VR group activity incorporating daily life tasks has shown to be a promising method for improving psychosocial capacities, including adherence to regulations, flexibility, and competence retention, among older adults in nursing homes.

#### Activity and Mobility

The results of the study demonstrate significant enhancement in the older adults’ proactivity (Mini-ICF-APP). This suggests that the older adults experience less boredom and express a desire to engage in more daily activities that interest them while living in the nursing home. They are better prepared and motivated to initiate activities on their own. Furthermore, the ADL score, particularly in the mobility domain, also showed a significant improvement. This improvement in proactivity can be linked to the enhanced mobility observed in the ADL scale. The nursing home already provides gymnastic courses and physical therapy to help older adults maintain or rebuild their physical functioning. When older adults are more willing to participate in these activities, it can lead to better mobility and daily activity (eg, using the toilet) outcomes, aligning with the theoretical background that meaningful group activities can improve activity and mobility in nursing homes. Previous studies have investigated the impact of VR interventions on ADLs and instrumental ADLs among older adults, yielding different results. One of these studies aligns closely with our own study. Liao et al [47] conducted a VR cognitive training program that involved tasks such as locating stores and acting as a virtual kitchen chef, which is similar to our approach. Their results demonstrated a significant improvement in older adults’ activity levels, with the effect size being larger than that observed with traditional cognitive training methods. Moreover, the improvement in the activity of older adults in nursing homes through VR intervention was also demonstrated by Saredakis et al [73]. In contrast, Optale et al [43] conducted a VR memory training program that consisted of repeated memory tasks focused on objects and orientation. Their study did not find a positive impact on ADL. This discrepancy suggests that the content of the VR intervention may play a crucial role in determining its effectiveness in enhancing older adults’ daily activities. Overall, these findings highlight the importance of considering the specific content and nature of VR interventions when assessing their potential impact on the daily lives of older adults.

On the basis of the exploratory findings of this study, conducting an experimental study to provide evidence about the impact of VR interventions on proactivity and mobility would be valuable. Such a study can further validate the potential benefits of VR interventions in promoting proactivity and improving mobility among older adults in nursing homes.

#### Group Integration and Social Interaction

There was significant improvement in the group capacity of the older adults, indicating their increased willingness to participate in group activities and enjoy the benefits of group engagement. Thus, VR intervention could be used as a meaningful group activity that contributes to reducing social isolation. Staff members in some care homes also reported that older adults were pleasantly surprised by the VR technology and were more open to group activities after participating in the VR project. These findings are consistent with the results reported by Fan et al [46], who conducted a VR intervention involving horticultural group activities such as gardening for community-dwelling older adults, aiming to reduce social isolation. In addition, other previous studies involving VR horticultural activities in nursing homes have also demonstrated a reduction in older adults’ loneliness and an improvement in their social interaction [44,74]. However, it is important to note that, unlike our study, these previous studies did not specifically focus on VR interventions as group events. Therefore, these studies have not reported about the impact of VR interventions specifically on group capacities of older adults. In addition to the VR horticultural activities implemented in nursing homes, the study by Saredakis et al [73] examined the effectiveness of VR reminiscence therapy in reducing older adults’ loneliness but did not observe significant effects. This suggests that VR horticultural activities may hold greater potential in reducing loneliness among older adults. Engagement in meaningful activities, such as virtual gardening or horticultural group activities, might have a more profound impact on addressing the issue of loneliness in this population.

It is important to note that the project spanned periods of the COVID-19 pandemic and the winter and summer seasons, which could potentially act as confounding factors. While there was generally limited availability of group activities during the pandemic, the introduction of VR group sessions may have enhanced older adults’ interest in social interaction.

#### Well-Being

Although there are statistically significant differences in the changes in well-being over the course of the intervention, it is important to note that the VR group activity has not yet demonstrated its full potential in consistently improving the well-being of older adults. The results indicate that there have been very small, incremental improvements in the well-being curve. The statistically significant difference observed may be attributed to the number of measurement points used in the study. We do not have a sufficient, practically relevant effect to confirm an increase in well-being.

The well-being of the older adults was already good at the beginning of the VR intervention study, which makes significant and consistent additional improvements less likely. Furthermore, well-being is influenced by various situational factors, particularly among older adults with health problems or disabilities considered vulnerable. Another factor to consider is the frequency and duration of the VR intervention, which may not have been sufficient to produce further improvement in well-being. Other VR studies that have demonstrated improvements in well-being often involve more frequent and longer VR interventions [44,50,74] or are only measured once after a 1-time intervention [48,75,76]. Furthermore, recent entertainment-oriented VR interventions targeting well-being or quality of life among older adults have predominantly used a passive interaction approach, for instance, virtual travel in Hong Kong [75]. These studies consistently achieved their research goals in terms of enhancing older adults’ mood and well-being. In contrast, VR interventions that primarily focus on functional training with hand interaction have generally shown limited improvement in overall well-being, such as the one conducted by Brito et al [77]. The learning process associated with using the hand console can act as a barrier for older adults, potentially hindering their ability to improve their well-being through functional training. Consequently, it is crucial to approach the didactic process of VR devices with care to ensure that older adults are not discouraged at the initial stages of training. Furthermore, when developing VR interventions for older adults, it is important to consider the design and usability of the console or device being used. Older adults may have specific needs and challenges when it comes to interacting with technology. Therefore, the console or device should be tailored to accommodate their physical abilities, cognitive capabilities, and potential sensory impairments [78].

In summary, the well-being of older adults could be maintained at a high level over the course of the VR group intervention. It would be interesting to see if a more frequent intervention could further improve the impact of VR intervention on the well-being of older adults.

### Strengths and Limitations

This study explored VR group activities in nursing homes, adopting a naturalistic approach to gain a deeper understanding of technology’s role for older adults in the digital age. Our findings revealed the potential of VR as a tool in meaningful activity programs for older adults residing in nursing homes. Notably, this intervention leads to an enhancement in older adults’ abilities and engagement in activities, while sustaining a high level of well-being. Our study offers novel insights into the transformative possibilities of VR for enriching the lives of older residents within nursing home settings. Despite the significant findings of this study, it is important to acknowledge several limitations.

First, the impact of the COVID-19 pandemic on the effectiveness of VR interventions cannot be ignored. During the pandemic, there were restricted group activities and increased vulnerability among older adults, which may have magnified the positive impacts of the VR group intervention. It is crucial to consider this unique context when interpreting the results.

Second, there may be a selection bias in the sample of participants. The selection of participants in the nursing home was based on defined and standardized selection criteria, which the nurses applied in the field. This is the most natural, accepted, and standardized way to select participants for psychosocial activities in nursing homes. There can be a slight selection bias due to the various individual interaction processes of the nurses with the participants. The selected sample may not accurately represent the range of responses in the population, but it represents older adults with complex disabilities who are nevertheless able to cope with specially designed VR tools. Moreover, recruitment was also based on older adults’ willingness to participate in the study. The older adults who chose to participate in the study may be more open to new experiences compared to those who declined. Furthermore, the findings indicate that the older adults initially reported good well-being and had regular contact with family or friends, suggesting a limited scope for improvement in well-being and social interaction. It is essential to find ways to extend the reach of VR group interventions to a wider range of older adults, particularly those who are more isolated and lonely. Using a VR session to introduce the intervention to these older adults may be a potential solution.

Third, there is a possibility that the older adults may have overestimated or underestimated their own capacities. This could be addressed by staff members closely monitoring the older adults’ daily behavior. However, due to limited personnel resources, this was not feasible in this study.

Finally, another weakness of this study is in its study design. This was not a randomized controlled trial. Although a control group was included, the sample size was very small, making it challenging to establish a valid comparison with the intervention group. Participation in the control group was based on the natural decisions of the older adults. Therefore, the results should be interpreted as a point of reference rather than indicative of a causal “effect.” Nevertheless, it is important to note that this longitudinal study is naturalistic and externally valid. It offers a novel perspective on the pragmatic application of VR intervention as a group event in nursing homes.

### Future Studies

On the basis of the findings of this exploratory study, a randomized controlled experimental trial that specifically focuses on VR group interventions within the daily lives of older adults in nursing homes should be conducted. Without the specific conditions during the COVID-19 pandemic, a more favorable social environment will be available, resulting in fewer hindering factors such as limited group interventions. In addition, in this study, large variation was observed in the basic cognitive functions of the participating older adults, according to age and type of disease. However, it is important for researchers to be mindful about the competencies and skill levels of older adults when introducing VR interventions [79]. Some older adults may feel socially excluded if they lack the necessary skills to participate in these digital activities [80]. Therefore, it is crucial to prioritize accessibility and provide adequate support and training to ensure inclusivity. This can involve tailoring the VR experiences to accommodate older adults with varying cognitive functioning, such as providing different levels of difficulty based on individual capabilities. In our study, we received diverse feedback from older adults regarding VR tasks. The highly independent older adults expressed that the VR tasks were very easy for them, whereas those with cognitive impairments or dementia found the tasks challenging to complete. In the next phase, it would be beneficial to group older adults based on their cognitive capacities and provide tailored VR interventions at different difficulty levels. Exploring an older adult–centered VR design is another intriguing direction for further investigation. This could involve studying the optimal form of interaction that minimizes the learning curve associated with using VR devices, ultimately enhancing the overall user experience for older adults.

### Conclusions

In conclusion, the project successfully explored the benefits of a VR-based group intervention in nursing home settings. The results indicate that the VR intervention could be a meaningful group activity in nursing homes to support social group interaction, activity level, and well-being. The 4 sessions of the VR group intervention—with tasks that the older adults were unable to perform in their current environment— led to significant improvements in adherence to rules, flexibility, competence, proactivity, group integration, and mobility. Future research could benefit from conducting a randomized controlled trial to provide stronger evidence.

## Appendix

Multimedia Appendix 1 Sociodemographic data of the participating older adults in the virtual reality intervention for the control group and the intervention group.

Multimedia Appendix 2 Comparison of the scores of the older adults from the control group and the intervention group at baseline, at the end of the virtual reality intervention, and 3 weeks after the postintervention assessment regarding their psychosocial capacities.

Multimedia Appendix 3 Comparison of the scores of the older adults from the control group and the intervention group regarding their well-being before the intervention (T0 and T1), during the intervention (T2-T5), after the intervention (T6), and 3 weeks after the postintervention assessment (T7).

## Abbreviations

ADL: activity of daily living
HMD: head-mounted device
Mini-ICF-APP: Mini-ICF-Rating for Impairment in Psychological Activities and Capacities
VR: virtual reality
WHO-5: World Health Organization–Five Well-Being Index

## Data Availability

The data sets generated during and analyzed during this study are available from the corresponding author on reasonable request.
