# Supplementary material for: Impact of Virtual Reality–Based Group Activities on Activity Level and Well-Being Among Older Adults in Nursing Homes: Longitudinal Exploratory Study
Source: JMIR Serious Games. 2024 Mar 29;12:e50796. doi: 10.2196/50796 (PMC11015370; doi:10.2196/50796)
Supplement: Multimedia Appendix 1 [file games_v12i1e50796_app1.docx]

**Appendix 1** Sociodemographic data of the participating seniors in the VR intervention for controlled group (CG) and intervention group (IG)

| Features |  | IG  (N = 116) | CG  (N = 12) | |
| --- | --- | --- | --- | --- |
| Gender | Female | 69.8% | | 83.3% |
|  | Male | 30.2% | | 16.7% |
| Age | Mean value | 80.74 | | 83.75 |
|  | SD | 8.49 | | 8.97 |
|  | Min / Max | 60-97 | | 61-94 |
| Education | None | 6% | | 25% |
|  | Special school | 1.7% | | 0% |
|  | Primary school | 0.9% | | 0% |
|  | Secondary school | 14.7% | | 41.7% |
|  | Primary school / 9th or 10th grade | 68.1% | | 33.3% |
|  | Abitur | 8.6% | | 0% |
| Professional qualification | None | 31.9% | | 25% |
|  | Craft profession. Skilled work | 57.8% | | 66.7% |
|  | Master | 6% | | 8.3% |
|  | University studies | 4.3% | | 0% |
| Longest professional activity in working life | Craft. Industry. Production | 31.9% | | 25% |
|  | Research and development | 2.6% | | 0% |
|  | Agriculture | 2.6% | | 0% |
|  | Office / Management activities | 20.7% | | 33.3% |
|  | Service. Gastronomy. Customer service | 22.4% | | 41.7% |
|  | Practical health care (nursing. doctor. therapist. or similar) | 8.6% | | 0% |
|  | Housewife | 10.3% | | 0% |
|  | Assisting family member | 0% | | 0% |
|  | Missing indication | 0.9% | | 0% |
| Frequent of visits from trusted people | Several times a week | 54.3% | | 66.7% |
|  | Weekly | 23.3% | | 0% |
|  | Every two to three weeks | 6.9% | | 16.7% |
|  | Monthly | 1.7% | | 0% |
|  | Less frequently than monthly | 1.7% | | 8.3% |
|  | No regular contacts | 12.1% | | 8.3% |
| Previous experience with VR | No | 92.2% | | ng |
|  | yes | 7.8% | | ng |
