# Supplementary material for: Impact of Virtual Reality–Based Group Activities on Activity Level and Well-Being Among Older Adults in Nursing Homes: Longitudinal Exploratory Study
Source: JMIR Serious Games. 2024 Mar 29;12:e50796. doi: 10.2196/50796 (PMC11015370; doi:10.2196/50796)
Supplement: Multimedia Appendix 2 [file games_v12i1e50796_app2.docx]

**Appendix 2** Comparison of the scores of the older adults from the control group and the intervention group at baseline, at the end of the virtual reality intervention, and 3 weeks after the postintervention assessment regarding their psychosocial capacities (Mini-ICF-Rating for Impairment in Psychological Activities and Capacities).

|  | **At the baseline (T0)** | | **At the end of the interventions (T6)** | | **3 weeks after the post-test (T7)** | | **IG**  **rANOVA (n =84)** | |
| --- | --- | --- | --- | --- | --- | --- | --- | --- |
|  | **IG (n = 84)** | **CG (n =11)** | **IG** | **CG** | **IG** | **CG** | ***p*** | **eta²** |
| **Capacities (Mini ICF APP)** |  |  |  |  |  |  |  |  |
| Adjustment to rules and routines | 2.57±0.88 | 2.09±,30 | 2.15±1.05 | 1.55±1.13 | 2.04±1.08 | 1.64±1.12 | <.001*** | .122 |
| Planning and structuring tasks | 3.13±1.82 | 2.64±1.36 | 2.95±1.92 | 1.64±1.75 | 3.13±2.08 | 1.91±1.70 | .557 | .007 |
| Flexibility and adaptability | 2.38±,90 | 1.82±1.17 | 1.86±1.19 | 1.00±,89 | 1.87±1.22 | 1.73±1.19 | <.001*** | .109 |
| Competence and knowledge application | 2.25±1.25 | 1.64±1.12 | 1.99±1.55 | 1.45±1.44 | 1.94±1.52 | 1.45±1.04 | .043* | .039^a^ |
| Capacity to make decisions and judgements | 2.57±1.12 | 2.09±,94 | 2.42±1.40 | 1.55±1.44 | 2.48±1.35 | 2.09±1.38 | .568 | .007 |
| Proactivity and spontaneous activities | 2.39±1.19 | 1.09±1.04 | 2.04±1.25 | 1.18±1.17 | 1.90±1.26 | 1.09±1.04 | <.001*** | .104 |
| Resilience and perseverance | 2.54±1.00 | 2.55±1.44 | 2.25±1.18 | 1.73±1.19 | 2.29±1.14 | 1.73±,65 | .066 | .032 |
| Self-assertiveness | 2.60±1.09 | 1.73±1.27 | 2.43±1.15 | 2.00±1.55 | 2.40±,96 | 2.36±1.36 | .303 | .014 |
| Capacity to talk and contact third parties | 2.39±1.41 | 1.64±1.12 | 2.14±1.35 | 2.09±1.76 | 2.25±1.42 | 1.45±1.44 | .149 | .023 ^a^ |
| Group capacity | 2.71±1.39 | 1.82±1.40 | 2.26±1.36 | 1.09±.83 | 2.04±1.21 | 1.64±1.21 | <.001*** | .141 |
| Capacity to form close relationships | 2.61±1.58 | 1.55±2.07 | 2.45±1.66 | 1.45±1.81 | 2.49±1.75 | 2.18±1.83 | .568 | .007 |
| Self-care and self-sufficiency | 3.29±1.76 | 3.73±2.33 | 3.18±1.84 | 2.45±2.58 | 3.11±1.86 | 3.45±1.69 | .625 | .006 |
| Mobility and transportability | 2.39±1.46 | 1.91±1.14 | 2.37±1.59 | 1.27±1.19 | 2.40±1.54 | 1.45±1.04 | .946 | .001 |
| Mini-ICF Mean | 2.60±.75 | 2.02±.73 | 2.35±.85 | 1.57±.94 | 2.33±.88 | 1.86±.58 | <.001*** | .150^a^ |
| **Activities of daily living (ADL),**  **total score** |  |  |  |  |  |  |  |  |
| Food | 9.29±1.76 | 10.00±0 | 9.46±1.56 | 10.00±0 | 9.64±1.30 | 10.00±0 | .112 | .027^a^ |
| Baden | 1.85±2.43 | 2.73±2.61 | 1.73±2.39 | 2.73±2.61 | 2.02±2.47 | 2.73±2.61 | .424 | .010 |
| Washing | 4.52±1.48 | 4.55±1.51 | 4.58±1.39 | 4.55±1.51 | 4.64±1.30 | 5.00±0 | .763 | .003 |
| Dressing and undressing | 7.20±3.41 | 7.73±2.61 | 7.38±3.68 | 7.73±2.61 | 7.32±3.51 | 7.27±3.44 | .837 | .002 |
| Stool control | 7.92±3.74 | 8.64±3.23 | 8.04±3.72 | 8.18±4.05 | 7.80±3.75 | 9.09±2.02 | .831 | .002 |
| Urine control | 6.31±4.40 | 6.82±4.05 | 6.55±4.25 | 6.36±4.52 | 6.90±4.31 | 7.27±3.44 | .270 | .016 ^a^ |
| Toilet use | 8.27±3.76 | 10.00±0 | 8.63±3.41 | 10.00±0 | 9.11±2.71 | 10.00±0 | .034* | .040 |
| Bed/(wheel)chair transfer | 12.74±4.93 | 15.00±0 | 13.15±4.58 | 15.00±0 | 12.98±4.66 | 15.00±0 | .217 | .018 ^a^ |
| Movement/Mobility | 9.11±4.40 | 9.09±3.75 | 9.46±4.39 | 9.55±4.16 | 9.64±4.16 | 9.55±2.70 | .035* | .039 |
| Climbing stairs | 4.35±4.40 | 5.00±4.47 | 4.76±4.17 | 5.91±4.91 | 4.88±4.46 | 6.36±5.05 | .284 | .015 |
| ADL total value | 71.55 ± 23.19 | 79.55±9.07 | 73.75± 23.32 | 80.00±15.00 | 74.94± 22.29 | 82.27±11.70 | .016* | .050 ^a^ |
