# Supplementary material for: Impact of Virtual Reality–Based Group Activities on Activity Level and Well-Being Among Older Adults in Nursing Homes: Longitudinal Exploratory Study
Source: JMIR Serious Games. 2024 Mar 29;12:e50796. doi: 10.2196/50796 (PMC11015370; doi:10.2196/50796)
Supplement: Multimedia Appendix 3 [file games_v12i1e50796_app3.docx]

**Appendix 3** Comparison of the scores of the older adults from the control group and the intervention group regarding their well-being (WHO-5: World Health Organization–Five Well-Being Index) before the intervention (T0 and T1), during the intervention (T2-T5), after the intervention (T6), and 3 weeks after the postintervention assessment (T7).

|  | **T0** | | **T1** | | **T2** | | **T3** | | **T4** | | **T5** | | **T6** | | **T7** | | **IG**  **rANOVA** | | |
| --- | --- | --- | --- | --- | --- | --- | --- | --- | --- | --- | --- | --- | --- | --- | --- | --- | --- | --- | --- |
| Items | IG (n=84) | CG (n) | IG (n) | CG (n) | IG (n) | CG (n) | IG (n) | CG (n) | IG (n) | CG (n) | IG (n) | CG (n) | IG (n) | CG (n) | IG  (n) | CG  (n) | F | *p* | eta² |
| Good mood, cheerfulness | 4.08±,95 | 4.36±,67 | 3.90±1.04 | 4.18±,87 | 3.78±1.13 | 4.09±,94 | 3.94±1.02 | 4.27±1.01 | 3.90±,99 | 3.91±,83 | 3.85±1.03 | 3.80±.92 | 3.83±.98 | 4.00±,89 | 3.90±1.04 | 4.18±.87 | 1.893 | .087 | -^a^ |
| Relaxation | 4.02±,01 | 4.36±,92 | 3.89±1.22 | 4.00±1.00 | 3.98±1.09 | 3.73±1.35 | 3.90±,96 | 4.00±1.34 | 3.94±1.04 | 3.45±1.21 | 3.93±1.02 | 3.80±1.14 | 4.06±1.09 | 3.91±,83 | 3.89±1.22 | 4.00±1.00 | .554 | .793 | .008 |
| Activity and energy | 3.54±1.27 | 3.73±,90 | 3.27±1.25 | 3.45±1.29 | 3.32±1.33 | 3.09±,94 | 3.52±1.21 | 3.55±,82 | 3.57±1.18 | 3.27±1.10 | 3.23±1.26 | 3.30±1.06 | 3.60±1.11 | 3.64±1.03 | 3.27±1.25 | 3.45±1.29 | 2.050 | .048* | .029 |
| Regenerative capacity through sleep | 3.49±1.35 | 3.91±1.58 | 3.65±1.36 | 4.09±1.22 | 3.71±1.26 | 4.55±1.21 | 4.03±1.05 | 3.73±1.68 | 4.03±1.21 | 3.36±1.29 | 3.89±1.36 | 3.90±1.66 | 4.01±1.12 | 3.55±1.37 | 3.65±1.36 | 4.09±1.22 | 3.867 | .001** | .054^a^ |
| Enthusiasm | 3.64±1.17 | 4.36±.67 | 3.72±1.28 | 4.55±,52 | 3.60±1.17 | 4.55±,69 | 3.73±1.22 | 4.18±.98 | 3.85±1.22 | 3.91±.83 | 3.68±1.29 | 4.00±,82 | 4.12±1.12 | 4.45±,82 | 3.72±1.28 | 4.55±.52 | 3.541 | .001** | .049 |
| WHO-5_Total | 3.75±,75 | 4.15±,64 | 3.69±,83 | 4.05±,68 | 3.68±,83 | 4.00±,63 | 3.82±,79 | 3.95±,82 | 3.86±,82 | 3.58±,87 | 3.72±,84 | 3.76±.84 | 3.92±.78 | 3.91±,65 | 3.91±.87 | 4.00±.65 | 2.235 | .041* | .032^a^ |
